# Tuberculosis outbreaks among students in mainland China: a systematic review and meta-analysis

**DOI:** 10.1186/s12879-019-4573-3

**Published:** 2019-11-14

**Authors:** Hongdan Bao, Kui Liu, Zikang Wu, Xiaomeng Wang, Chengliang Chai, Tieniu He, Wei Wang, Fei Wang, Ying Peng, Bin Chen, Jianmin Jiang

**Affiliations:** 1 Medical Insurance Management Office, Zhejiang Hospital, Hangzhou, 310013 Zhejiang China; 2 Zhejiang Provincial Centers for Disease Control and Prevention, Hangzhou, 310051 Zhejiang China; 3 Research office, Women’s Hospital School of Medicine, Zhejiang University, Hangzhou, 310006 Zhejiang China; 4 Zhejiang provincial Centers for Disease Control and Prevention, Hangzhou, 310051 Zhejiang China; 5 Key laboratory of Vaccine, Prevention and Control of infectious disease of Zhejiang prevince, Hangzhou, 310051 Zhejiang China

**Keywords:** Tuberculosis, School, Outbreak

## Abstract

**Background:**

In recent years, tuberculosis outbreaks in schools have occurred more frequently in China than in other parts of the world, and have posed a public health threat to students and their families. This systematic review aimed to understand the epidemiological characteristics of tuberculosis (TB) outbreaks and analyze the factors associated with TB outbreaks in schools in China.

**Methods:**

We conducted this systematic review following the standard procedures of the Cochrane Collaboration and the Preferred Reporting Items for Systematic Review and Meta-Analysis statement. The meta-analysis was performed with STATA using a random effects model.

**Results:**

We included 107 studies involving 1795 student patients with TB in mainland China. The results of the systematic analysis indicated that TB outbreaks were more frequently reported in senior middle schools and in Eastern China. The outbreaks mainly occurred during the winter and spring, and the median outbreak duration was 4 months. The meta-analysis showed that the total attack rate and the class attack rate of tuberculosis outbreaks among students were 4.60% (95% CI 3.80 to 5.70%) and 22.70% (95% CI 19.20 to 27.00%), respectively. Subgroup analysis showed that outbreaks that occurred at universities or colleges had a relatively higher attack rate than those occurred in senior middle schools. The prevalence of latent tuberculosis infection (LTBI) among close contacts was 23.70% (95% CI 19.50 to 28.90%). The median case-finding interval was 2 months, and 47.40% of the index cases had a case-finding delay.

**Conclusion:**

The results of our review indicated that school TB outbreaks were reported most frequently in senior middle schools in China. The attack rates of outbreaks at universities or colleges were higher than those in senior middle schools. The TB outbreaks in schools usually occurred over prolonged periods. The case-finding delay in the index cases must be reduced to prevent transmission in classes and schools. Effective surveillance and screening of presumptive TB cases in schools should be strengthened to reduce outbreaks in schools.

## Background

Tuberculosis (TB) is one of the top 10 causes of death worldwide [1, 2]. In 2017, 10.1 million people fell ill with TB, and 1.6 million of them died from the disease (including 0.3 million people with HIV). About 87% TB cases was from 30 countries with the heaviest burden of tuberculosis [3]. China is one of 30 countries with the heaviest burden of tuberculosis patients, and it has the third highest number of cases [3, 4]. According to the fifth national tuberculosis epidemiological survey conducted in 2010, the prevalence of active pulmonary TB and smear-positive pulmonary TB was 459/100,000 and 66/100,000, respectively, in the population aged over 15 years [5]. In 2012, the reported incidence of PTB was 16.63 per 100,000 students [6]. According to the China Information System for Disease Control and Prevention, student TB patients accounted for 4.02% of total TB patients in 2014 [6]. The top five provinces had the highest TB incidence rates among school students, including Tibet(79.95/100,000), Qinghai(59.09/100,000), Guizhou(36.54/100,000), Chongqing(33.06/100,000) and Xinjiang(26.08/100,000) [7, 8], which are mainly western regions of china. As a respiratory infectious disease, PTB is more likely to spread within clusters and subsequently progress to outbreaks. According to previous studies, school TB outbreaks, refer to 3 or more cases with an epidemiological link at the same school within 2 years [9, 10]. The epidemiological link refers to the clustering of cases with a mutual contact history as detected by a field investigation in one place during the outbreak period [11]. Pulmonary TB outbreaks in schools usually cause an enormous social impact. According to a study investigating an outbreak in a training school in Shanxi Province, the prevalence of TB was 15.70%, which was a serious clustered outbreak that lead to an irreversible impact on students [12]. The recent outbreak of school tuberculosis in Taojiang County, Hunan Province was another typical example. According to related news reports, 81 confirmed tuberculosis cases and 7 presumptive tuberculosis cases were reported in this incident [13]. The outbreak caused a substantial social impact, especially on students and their families. Therefore, once tuberculosis spreads widely among students, it causes great panic among classes and schools and even affects social stability.

China has a relatively comprehensive mechanism for reporting school TB outbreaks. The outbreaks are reported to different institutions according to the number of cases. Outbreaks with more than 3 but fewer than 10 cases are reported to the local government, while outbreaks with more than 10 cases are reported to the government over province level as an emergency public health event. Once the Centers for Disease Control and Prevention (CDC) detects 3 or more cases based on the surveillance system, the Health and Education Department should investigate and address the epidemic, mainly through close contact screening, tracing the sources of infection, etc. An outbreak investigation report is then written by the Health Department and submitted to the government.

To the best of our knowledge, most research articles are case reports of single-school TB outbreaks in China. However, it is difficult to systematically obtain information about the epidemiological features of school TB outbreaks from single outbreak case reports. No studies have systematically reviewed TB outbreaks among Chinese schools. It is important to synthesize the characteristics of TB outbreaks among schools to help develop public health strategies for school TB outbreaks.

We conducted this systematic review and meta-analysis to identify all the available published case studies in the Chinese and English languages that reported TB outbreaks among schools in mainland China. The objectives of this review are the following: i) to obtain the attack rate of TB and prevalence of LTBI among students during TB school outbreaks in China; ii) to understand the main characteristics of school TB outbreaks, including the age and sex distribution of cases, outbreak duration, characteristics of the index case, and intervention measures; and iii) to analyze the factors associated with TB outbreaks in schools in China.

## Methods

### Search strategies

We searched the following six English and Chinese electronic databases for primary studies: China Knowledge Resource Integrated Database (http://www.cnki.net/); Wanfang Med Online (http://med.wanfangdata.com.cn/); Chinese Biomedical Literature Database(CBM) (http://www.sinomed.ac.cn/); VIP database (http://lib.cqvip.com/), PubMed (https://www.pubmed.gov); ScienceDirect, which is Elsevier’s leading platform for peer-reviewed scholarly literature (http://www.sciencedirect.com/search?qs=); and Web of Science (http://isiknowledge.com). Our search strategy included terms such as “tuberculosis”, “outbreak”, “school”, “university”, and “college” (details of the complete search strategy are provided in Additional file 1). All the results were retrieved in June, 2019. This systematic review and meta-analysis followed the Preferred Reporting Items for Systematic Reviews and Meta-Analyses guidelines [14].

### Inclusion and exclusion criteria

The inclusion criteria were 1) outbreaks that occurred at schools (junior middle schools, senior middle schools and universities, ≥3 reported cases with an epidemiological link; 2) sufficient basic information (school type, school location and epidemiological investigation information regarding the outbreak); 3) clear diagnosis of tuberculosis; and 4) epidemiological investigation processing of the outbreak (tracing of the index case, a clear contact investigation process, epidemiological analysis of cases and intervention measures). The exclusion criteria were 1) the research article was a review or described a nonoutbreak; 2) the reports and studies lacked key information on the epidemiological investigation process; and 3) the TB outbreaks occurred before 2000.

### Data extraction

Two reviewers independently screened the citations (titles and abstracts) identified from all sources. The data extracted from the studies included the following items: author, publication year, journal, title, outbreak duration, outbreak area, school type, the living accommodation space of one person, attack rate, prevalence of LTBI, screening criteria, number of different TB types, interventions, and information on the index cases (age, sex, grade, time of TB onset, case-finding interval, secondary case onset time, and clinical information such as TB type and the results of sputum smears and chest X-rays). The terms used in this study are defined below.

School: included three types: i) junior middle school and primary school (aged 6–15 years), China’s 9-year compulsory education for all citizens [15]; ii) senior middle school (aged 15–17 years), the continuation of junior middle school education considered to be a critical preparation for college education [16]; and iii) university or college (age above 17 years), defined as an institution of higher education offering education in mainly nonvocational subjects and typically having the power to confer degrees [17]. University or college education usually lasts 4 years in China.

Outbreak: ≥3 epidemiological linked cases within 2 years, based on the CDC guidelines for contact investigations [9, 10].

Outbreak duration: the time period between index case confirmation and final case confirmation.

Attack rate: an incidence within a limited area during a short period [18]. In this study, we defined two attack rates, namely, the total attack rate and the class attack rate. The former rate was based on the screened populations and, was calculated as the number of new cases divided by the number of the screened populations. The number of the screened population was determined by the number of close contacts of the index case during an outbreak; these close contacts might have been from one class, several classes or the entire school. The class attack rate was based on the classes where the index cases occurred, calculated as the number of new cases in each class divided by the number of students in that class.

Prevalence of LTBI (latent tuberculosis infection) and screening criteria: LTBI refers to individuals who are infected with *Mycobacterium tuberculosis*, but do not develop active tuberculosis. Students who had a strong positive PPD (purified protein derivatives tuberculin) result were identified as LTBI. A strong positive result mainly refers to the judgment criteria used in different studies, namely, induration size more than 15 mm or 20 mm [19, 20], depending on the study. Articles used chest radiography as a screening method in our calculation of the prevalence of LTBI were excluded. In addition, we defined two prevalence of LTBI, namely, the total prevalence of LTBI and the prevalence of LTBI among close contact. The former was calculated as the number of students with LTBI divided by the number in the screened population, which may not be the close contacts of the index cases (all of school students may be screened in some outbreaks). The prevalence of LTBI among close contact was calculated as the number of students with LTBI divided by the number of close contacts who were strictly confirmed.

Season when the index case was detected: the season was divided according to the Meteorological Department of China. Spring occurred from March to May, summer occurred from June to August, autumn occurred from September to November, and winter occurred from December to February.

Case-finding interval: the time interval between the onset of TB symptoms and the first diagnosis of TB.

Case-finding delay: a case-finding interval longer than 2 months [19].

Index case: the first case with TB symptoms detected by field epidemiological investigation in one outbreak.

### Data analysis

The meta-analysis was performed using STATA version 14.0 for Windows (STATA Corporation, College Station, Texas, United States). A random effects model was used for the analysis. The results are presented in tables (rate, number of studies, 95% CI, heterogeneity) and forest plots. Publication bias was assessed by a funnel plot. The systematic review was performed using Statistical Package for Social Sciences (SPSS) version 18.0 (SPSS Inc., Chicago, Illinois, USA). We used meta-regression to explore the associations among variables.

## Results

### Identified studies

**Fig. 1:**
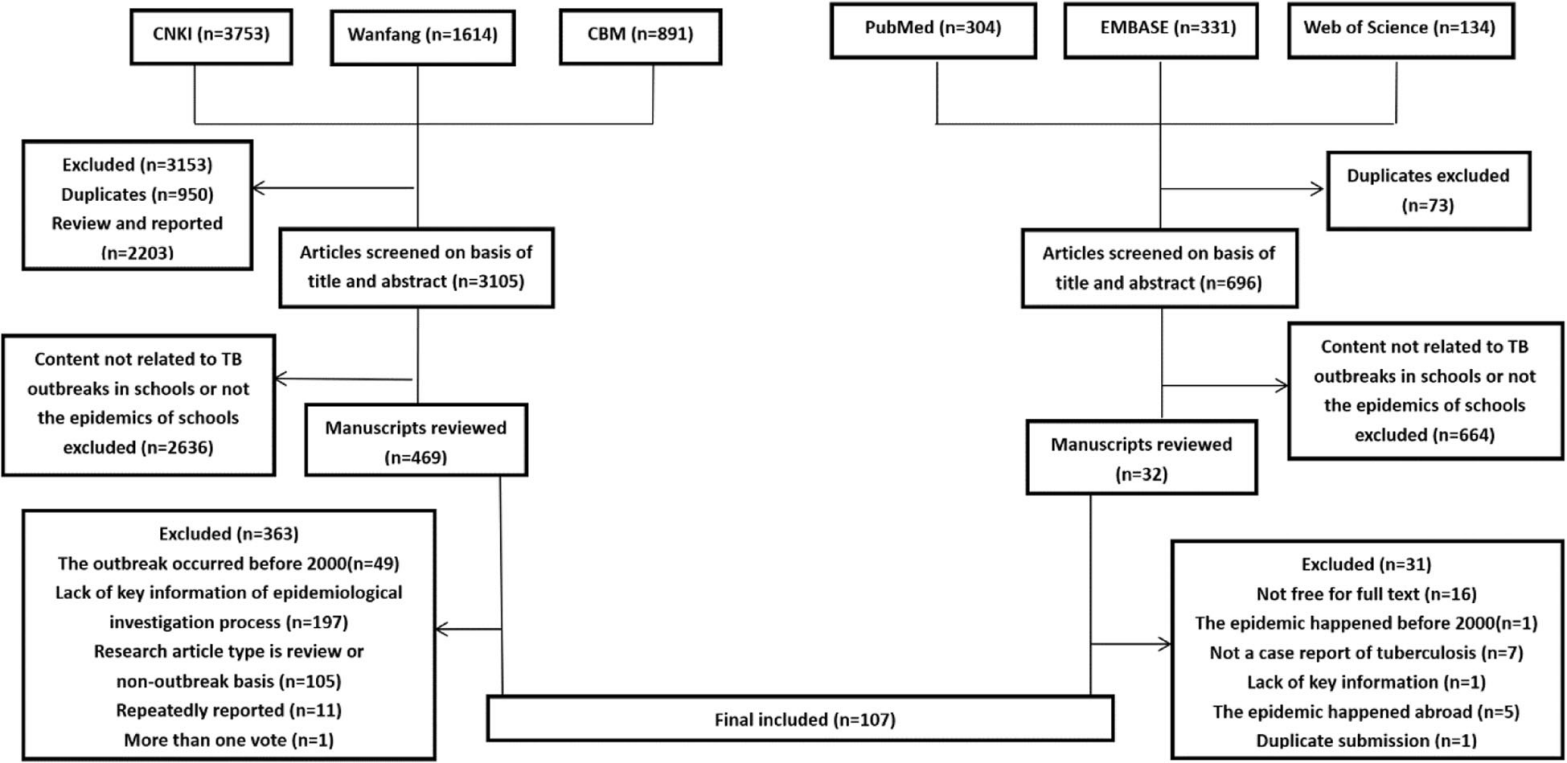
Flow diagram of the literature search and study selection

Among the 7027 articles we searched in the databases, 107 articles met our inclusion criteria (Fig. 1) [21–127]. A total of 79 (73.80%) studies reported the attack rate of TB, and 81 (75.70%) studies determined the prevalence of LTBI. Additionally, 80 articles reported the interventions for the outbreaks.

**Fig. 2:**
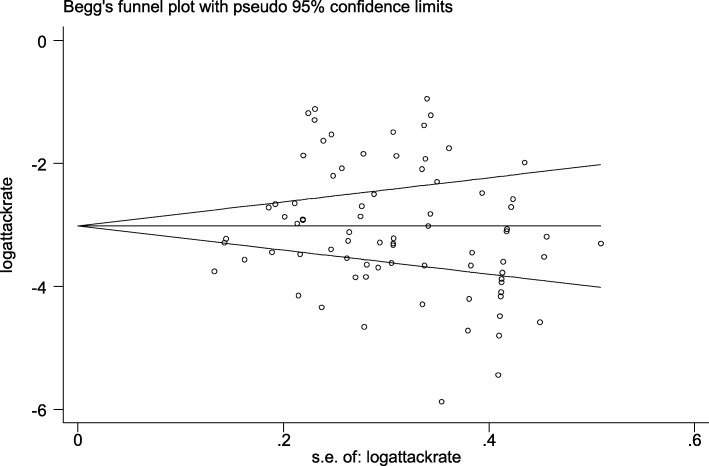
Funnel plot of the total attack rate school TB outbreaks

Publication bias was tested with Begg’s test. The results showed that the *P* value was 0.34, which indicated that there was no significant publication bias (Fig. 2) [21–127].

### Main results

#### Outbreak and patient summaries

The 107 outbreaks included in our review involved 1795 student TB patients (median 16.8 cases/outbreak, range 4–66 cases) [21–127]. Among these outbreaks, 66 (61.70%) outbreaks occurred at senior middle schools and involved 1100 patients; universities or colleges followed, with 29 (29.00%) outbreaks involving 587 patients. Only 10 outbreaks occurred at junior middle schools or primary schools and involved 108 patients. There were 22 (20.60%) outbreaks that lasted less than 1 month, and 34 outbreaks lasted more than 4 months. In terms of geographical distribution, there were 78 (72.90%) outbreaks involving 1207 patients reported in the eastern provinces of China while 13 (12.10%) outbreaks occurred in central provinces. As for the time season of index case detection, most outbreaks occurred in winter, accounting for 41.10% (Table 1).

**Table 1:** Characteristics of 107 outbreaks among students

Characteristic	Number of studies	Percent (%)
Type of school		
Junior middle school or primary school	10	9.30
Senior middle school	66	61.70
University or college	31	29.00
Outbreak duration		
1 month or less	22	20.60
1–2 months	24	22.40
2–4 months	27	25.20
4 months or longer	34	31.80
Region where the outbreak school was located		
Eastern region	78	72.90
Central region	13	12.10
Western region	16	15.00
The season when index case was detected		
Spring	30	28.00
Summer	17	15.90
Autumn	16	15.00
Winter	44	41.10

Except for 484 patients whose sex was not documented, 799 (60.95%) cases were males, and 512 (39.05%) cases were females. Among the 1795 patients, 1572 (90.40%) patients had pulmonary TB, including 263 (16.73%) with smear-positive TB and 1309 (83.27%) with smear-negative TB. Another 167 (9.60%) patients had had extrapulmonary tuberculosis. There were 393 cases that reported a tuberculous cavity, of which 37 (9.41%) patients had cavernous pulmonary tuberculosis. Additionally, among LTBI cases in the studied outbreaks, 2398 students had received prophylaxis, while only 1645 (68.59%) finished 6 months of prophylaxis (Table 2).

**Table 2:** Characteristics of 1795 student TB patients in 107 studies

Characteristic	Number of cases	Percentage (%)
Type of outbreak school		
Junior middle school	108	6.00
Senior middle school	1100	61.30
University or college	587	32.70
Sex		
Male	799	60.95
Female	512	39.05
Data not reported	484	----
Region where the outbreak school was located		
Eastern region	1207	67.20
Central region	273	15.20
Western region	315	17.50
TB classification		
Pulmonary tuberculosis	1572	90.40
Extrapulmonary tuberculosis (including Tuberculous pleurisy)	167	9.60
Data not reported	56	----
Sputum smear		
Negative	1309	83.27
Positive	263	16.73
Data not reported	228	----
Cavernous pulmonary tuberculosis		
Yes	37	9.41
No	356	90.59
Data not reported	1402	----
Students with LTBI who had received prophylaxis		
Treatment completed	1645	68.59
Treatment not completed	753	31.41

---- no data in the cell

### Characteristics of the index cases

Table 3 presents the characteristics of the index cases of the outbreaks. According to 72 studies reporting the gender of the index cases, 54 (75.00%) cases were males, and 18 (25.00%) were females. A total of 17 outbreak studies reported the family history of the index case, among which 7 cases had a family history. A total of 90 outbreak studies provided information on sputum smear results, among which 75 (83.30%) cases were positive, and 15 (16.70%) cases were negative. There were 20 outbreak studies that reported the degree of smear positivity. The median case-finding interval was 2 months. A total of 48 (53.93%) cases had a case-finding interval less than or equal to 2 months, while 45 (47.40%) cases had a case-finding interval longer than 2 months, which was defined as a case-finding delay. The median interval from the confirmation of the first case to the report of the second case was 4 months among 37 outbreaks.

**Table 3:** Characteristics of the index case

Characteristic	Number of studies	Number of patients	Percentage (%)
Sex	72		
Male		54	75.00
Female		18	25.00
Family history	17		
Yes		7	41.20
No		10	58.80
Type of school	107		
Junior middle school		10	9.30
Senior middle school		66	61.70
University or college		31	29.00
Sputum smear	90		
Positive		75	83.30
Negative		15	16.70
Smear positive	20		
1+		5	25.00
2+		6	30.00
3+		4	20.00
4+		5	25.00
Case-finding interval	95		
2 months or less		50	52.60
More than 2 months		45	47.40
Cavernous pulmonary TB	20	20	----

---- no data in the cell

### Attack rate of TB among students in mainland China, 2000–2017

As shown in the Tables 4, 79 (73.80%) studies determined the attack rate [21–92, 122–127]. The results showed that the pooled total attack rate of TB was 4.60% (95% CI 3.80 to 5.70%). Subgroup analysis suggested that the pooled total attack rate of junior middle schools was 5.80% (95% CI 2.80 to 12.80%), followed by universities or colleges with a pooled attack rate of 5.00% (95% CI 3.50 to 7.10%). The pooled attack rate of outbreaks at senior middle schools was 4.30% (95% CI 3.30 to 5.80%). The results showed that the pooled total attack rate among outbreaks with 10 or more cases was 6.10% (95% CI 4.70 to 7.20%), and the total attack rate in outbreaks with fewer than 10 cases was 3.00% (95% CI 2.10 to 4.30%). In terms of regions, the pooled attack rate of outbreaks in the eastern, central and western regions of China were 4.70% (95% CI 3.70 to 6.00%), 8.70% (95% CI 5.80 to 13.20%), and 2.80% (95% CI 1.70 to 4.90%), respectively(see details in Additional file 2).

**Table 4:** Meta-analysis of the total attack rates among TB students (*n* = 79)

Subgroup analysis	Attack rate (95% CI)	Number of studies	Heterogeneity	
			I^2^ (%)	*p*
Outbreaks classified by case number				
10 or more	6.10(4.70,7.20)	47	93.00	< 0.05
Fewer than 10	3.00(2.10,4.30)	32	86.30	< 0.05
Type of outbreak school				
Junior middle school	5.80(2.80,12.80)	5	85.40	< 0.05
Senior middle school	4.30(3.30,5.80)	48	91.90	< 0.05
University or college	5.00(3.50,7.10)	26	92.30	< 0.05
Region where the outbreak school was located				
Eastern region	4.70(3.70,6.00)	64	91.70	< 0.05
Central region	8.70(5.80,13.20)	6	71.40	< 0.05
Western region	2.80(1.70,4.90)	9	91.10	< 0.05
Overall effect	4.60(3.80,5.70)	79	91.70	< 0.05

Table 5 shows that the pooled class attack rate of 61 outbreaks was 22.70% (95% CI 19.20 to 27.00%) [21–25, 28, 29, 32, 36, 39, 44, 47, 50, 53, 55, 57, 59, 61, 63, 68, 70, 72, 74, 77, 81, 83, 86, 91, 93, 95, 99, 102, 104, 106, 108, 114, 115, 117, 122, 124–128]. Subgroup analysis showed that the pooled class attack rate for the 40 senior middle school outbreaks was 21.80% (95% CI 17.40 to 27.40%), and that of the 16 university or college outbreaks was 25.00% (95% CI 18.30 to 34.10%). In terms of geographic distribution, the pooled class attack rates for the eastern and central regions, almost the same, were 21.80% (95% CI 18.00 to 26.30%) and 22.00% (95% CI 11.70 to 41.30%), respectively. The western region showed a relatively high attack rate of 30.70% (95% CI 18.10 to 52.30%). The pooled class attack rate of outbreaks with an index case case-finding interval of 2 months or less was 22.00% (95% CI 17.30 to 28.00%), and the rate for outbreaks with an index case case-finding interval of more than 2 months was 22.50% (95% CI 17.30 to 29.30%). The data were further divided into subgroups based on the number of cases in the outbreak. The class attack rate of outbreaks with 10 or more cases was 30.50% (95% CI 25.30 to 36.70%), and that of outbreaks with fewer than 10 cases was 13.40% (95% CI 11.10 to 16.20%) (see details in Additional file 3).

**Table 5:** Meta-analysis of the class attack rate among TB students

Subgroup analysis	Prevalence (95% CI)	Number of studies	Heterogeneity	
			I^2^ (%)	*p*
Outbreaks classified by case number				
10 or more	30.50(25.30,36.70)	38	66.00	< 0.05
Fewer than 10	13.40(11.10,16.20)	23	2.70	0.42
Type of outbreak school				
Junior middle school	22.10(15.30,31.90)	5	14.80	0.32
Senior middle school	21.80(17.40,27.40)	40	73.40	< 0.05
University or college	25.00(18.30,34.10)	16	64.00	< 0.05
Region where the outbreak school was located				
Eastern region	21.80(18.00,26.30)	49	67.10	< 0.05
Central region	22.00(11.70,41.30)	5	79.70	< 0.05
Western region	30.70(18.10,52.30)	7	75.50	< 0.05
Case-finding interval of index cases				
2 months or fewer	22.00(17.30,28.00)	28	73.40	< 0.05
More than 2 months	22.50(17.30,29.30)	24	66.20	< 0.05
Overall effect	22.70(19.20,27.00)	61	69.00	< 0.05

### Prevalence of LTBI among students in mainland in China, 2000–2017

The pooled total prevalence of LTBI in 81 outbreaks was 20.50% (95% CI 16.90 to 24.80%). Subgroup analysis showed that the pooled total prevalence of LTBI in the group (induration≥15 mm) was 22.80% (95% CI 17.00 to 30.60%), and that of the group (induration≥20 mm) was 18.60% (95% CI 14.40 to 24.00%). Additionally, the pooled total prevalence of LTBI in outbreaks with 10 or more cases was 17.40% (95% CI 12.00 to 25.20%), and the total prevalence of LTBI in outbreaks with fewer than 10 cases was 22.20% (95% CI 17.70 to 27.90%).

The pooled prevalence of LTBI among close contacts in 73 outbreaks was 23.70% (95% CI 19.50 to 28.90%). Subgroup analysis showed that the pooled prevalence of LTBI among close contacts of the group (induration≥15 mm) was 27.60% (95% CI 20.40 to 37.30%), and that of the group (induration≥20 mm) was 21.70% (95% CI 16.10 to 29.40%). Furthermore, the pooled prevalence of LTBI among close contacts in outbreaks with 10 or more cases was 27.50% (95% CI 21.80 to 34.80%), and the prevalence in outbreaks with fewer than 10 cases was 18.80% (95% CI 13.50 to 26.20%).

### Factors associated with TB outbreaks, 2000–2017

Meta regression showed that the type of outbreak school, the region where the outbreak school was located, and the outbreak duration were correlated with the total attack rate of outbreaks (B_1_ = 0.37, p_1_ < 0.05; B_2_ = -0.31, p_2_ < 0.05, B_3_ = 0.22, p_2_ < 0.05); the type of outbreak school and the outbreak duration were correlated with the prevalence of LTBI (B_4_ = -0.34, p_4_ < 0.05; B_5_ = 0.16, p_5_ < 0.05); and only the outbreak duration was correlated with the class attack rate (r_6_ = 0.24, p_6_ < 0.01).

### Interventions for TB outbreaks, 2000–2017

We identified five main interventions for TB outbreaks. Eighty 80 (74.80%) outbreaks were addressed with an intervention that used standardized management and treatment of confirmed cases and implemented a school strict rehabilitation system. A total of 75 (70.10%) outbreaks conducted PPD testing and X-ray examinations of close contact students and provided prophylaxis for strongly positive students. Ventilating the classroom and dormitory more frequently and timely disinfecting were reported in 73 (68.20%) outbreaks. A total of 70 (65.40%) outbreaks carried out health education for teachers and students through health education courses, publicity panels, blackboard newspapers, leaflets and other methods. Moreover, 33 (30.80%) outbreaks took measures that involved monitoring and reporting the TB epidemic, strictly implementing morning inspections, tracking absenteeism due to the disease, and reporting cases in a timely manner.

## Discussion

This study was the first review to systematically analyze the epidemiological characteristics of school TB outbreaks in China. The pooled total attack rate of Chinese school TB outbreaks in the most recent 17 years was 4.60%, with 16.8 cases per outbreak. The pooled total prevalence of LTBI was 20.50% among the screened population. The pooled class attack rate was higher, at 22.70%. The median duration of outbreaks was 3 months, with a range of 1 month to 18 months. Additionally, the median case-finding interval for index cases was 2 months, which was higher compared with other studies [121, 129, 130].

In our meta-analysis, the attack rate was approximately much higher than the prevalence of active PTB according to the Fifth National Tuberculosis Epidemiology Survey and the average level of prevalence of student PTB in China [5, 8]. According to the 2018 TB report, the total TB incidence among general population in China was 88.9/100,000, which are also lower than the pooled attack rate reported in our review [3]. The high attack rate was probably due to the exposure to source of the infection in a closed environment. In addition to schools, there were also many other places with reported TB outbreaks, such as prisons and factories. According to research conducted in China and abroad, the prevalence of tuberculosis in prison is lower than that in outbreak schools included in our review [131–134] but higher than that among the general population [5]. All the results indicated that there was a high risk of TB in the closed places and clustering groups. More measures should be taken to reduce the risk of TB infection in such settings. The pooled prevalence of LTBI among close contacts (27.60%, TST ≥ 15 mm) was higher than the prevalence of LTBI among the general population worldwide (23%) [135] and in rural areas (19%, TST ≥ 15 mm) [136]. The prevalence of LTBI among school TB outbreaks was still higher than the study conducted by WL Meng, who reported a prevalence of 25.8% (TST ≥ 15 mm) over 5 outbreaks [137]. Furthermore, the prevalence of LTBI among students in our review was also higher than that reported in other countries [138–141]. For example, E. G. Teixeira et al demonstrated that the prevalence of LTBI among undergraduate students in Italy was 6.9% (TST ≥ 10 mm) [141]—far lower than our results. The basic TB burden in China was much heavier than Italy may partially explain the inconsistency [142, 143]. The meta-analysis also presented that the pooled total attack rate appeared to be higher than the attack rate in the eastern provinces or cities of China such as Beijing and Dalian [137, 144].

According to previous studies, the prevalence of active pulmonary tuberculosis in western provinces of China was higher than that in eastern provinces of China [5, 145]. Therefore, the higher attack rate of school TB outbreaks in the western region might result in a higher total attack rate among general population. However, we also found that far more outbreaks reported in eastern China than that in western China. This difference could be explained by the fact that researchers in the eastern provinces of China published articles more frequently than researchers in the western provinces. Notably, our estimate of the attack rate at universities was higher than that at senior middle schools, while the number of patients was highest at senior middle schools.. The outbreaks at senior middle schools mainly involved students in grade 12, who have the intensive burden of studying to prepare for college entrance tests [146, 147]. Hence, target actions should be taken for outbreaks at senior middle schools and universities. Our review also found that winter and spring seemed to be the two seasons for more frequent student TB outbreaks. The spring and winter peaks of student tuberculosis outbreaks could be the result of long-term contact in poorly ventilated rooms [148, 149].

Tracking and investigating index cases is a significant part of an epidemiological investigation of an outbreak. The results showed that the M:F ratio of all index cases was 3:1, which was much higher than that reported by the WHO and other studies [3, 150]. However, the M:F ratio among all student TB patients in our review was approximately 1.59:1, far lower than that of the index cases. This finding suggest that male students are more likely to be a potential source of infection because men have poorer health-seeking behaviors than women [151]. However, when males and females are exposed to the same source of an infection within the same closed space, the gender difference in TB susceptibility was not so obvious.

The case-finding interval of the index case is a significant factor in attack rate [152]. Through a subgroup analysis of the class attack rate based on the case-finding interval, we discovered that patients with a case-finding delay showed a relatively high class attack rate compared to those without a case-finding delay. A longer interval leads to greater opportunity of TB infection in the same space. Moreover, patients who were delayed in detection were more likely to develop severe pulmonary tuberculosis and increase the susceptibility among close contacts [121, 130]. The median time interval from the report of the first case to the emergence of the secondary case was 4 months, which indicated that this period could be recognized as a time window for timely interventions to prevent the occurrence of secondary cases.

According to the patient summary in our review, we found that only 16.70% of cases were reported to be sputum smear positive, while 83.30% of index cases were sputum smear positive. This finding suggests that the index cases were more contagious than subsequent cases. Therefore, it is necessary to isolate the source of the infection in a timely manner [153, 154]. Complete prophylaxis could greatly reduce the possibility of the subsequent activation and spread of tuberculosis [155, 156]. In our review, a total of the 2398 close contacts with strongly positive PPD test results had received prophylaxis, only 68.59% finished the six-month prophylaxis. The results also showed that there remains a shortage of knowledge concerning TB prevention and treatment among students. Therefore, health education regarding prophylaxis should be strengthen in more effective ways to ensure they exactly completed the six-month course of therapy.

We found a positive correlation between the outbreak duration and the TB attack rate, the prevalence of LTBI and class attack rate, which indicates that longer-lasting epidemics result in a more infectedstudents in the outbreak. Furthermore, we found that in areas from east to west, the prevalence of LTBI decreased while as previous studies have reported, the burden of tuberculosis is heavier in Western China than in Eastern China [157, 158]. A possible explanation for this result is that the sample size was too small when assessing the prevalence of LTBI in Western China.

We synthesized the interventions for school TB outbreaks that were reported in the included studies. Our review showed that few outbreaks (33/107) involved interventions that monitored the TB epidemic and implemented strict morning inspections, which suggests that schools should strengthen their epidemic monitoring and disease absence tracking abilities.

## Limitations

Our review had some limitation. Firstly, due to the geographical limitation of the search area on the mainland of China, the results may not be extrapolated to other regions and continents. Secondly, apart from the studies that our review included or excluded, there are still many outbreaks not published, especially in the western parts of China. This lack of coverage may have partially affected our conclusions. Moreover, some of the included studies had incomplete information concerning outbreaks led to information loss. Thirdly, most of the articles we included in our review were case reports from different settings and the infection source from some open universities might be multiple which may cause heterogeneity. Finally, due to the equipment inadequacy and technical deficiency of low level TB laboratories over the last decade in China, there were different diagnostic methods used among the included studies. Although we conducted subgroup-analysis, meta-regression to explain and reduce heterogeneity, it’s still hard to avoid.

## Conclusion

In conclusion, school TB outbreaks were more frequently reported at senior middle schools in China. The attack rates of outbreaks at universities or colleges were higher than those at senior middle schools, and the attack rate of class contacts was higher in the western provinces of China than that in the eastern provinces of China. Longer duration of case-finding was correlated with the severity of the outbreak. There was an urgent need to reduce case-finding delays in index cases to reduce the spread in classes and schools. Relevant departments and institutions should strengthen active case-finding measures such as monitoring and screening for presumptive TB cases in schools in order to prevent school outbreaks in early stage.

## Supplementary information

**Additional file 1: Table S1.** The search strategy of our review.

**Additional file 2: Figure S1.** Forest plot of total attack rates of different schools, **Figure S2.** Forest plot of total attack rates of different regions where the outbreaks schools located and **Figure S3.** Forest plot of total attack rates of different case number.

**Additional file 3: Figure S4.** Forest plot of class attack rates of different schools, **Figure S5.** Forest plot of class attack rates of different regions where the outbreaks schools located, **Figure S6.** Forest plot of class attack rates of different case number, **Figure S7.** Forest plot of class attack rates of different diagnose interval of index cases.

**Additional file 4:** **Table S2.** The outcomes of each outbreak for the meta-analysis.

## Data Availability

The key information generated and/or analyzed during this study are included as the additional files in this publication.

## Abbreviations

CDC: Centers for Disease Control
CI: Confidence interval
LTBI: Latent tuberculosis infection
M:F: Male:female
PPD: Purified protein derivatives tuberculin
PTB: Pulmonary tuberculosis
TB: Tuberculosis
TST: Tuberculin skin test
