# Supplementary material for: Tuberculosis outbreaks among students in mainland China: a systematic review and meta-analysis
Source: BMC Infect Dis. 2019 Nov 14;19:972. doi: 10.1186/s12879-019-4573-3 (PMC6854678; doi:10.1186/s12879-019-4573-3)
Supplement: Supplementary file 1 — Additional file 1: Table S1. The search strategy of our review. [file 12879_2019_4573_MOESM1_ESM.pdf]

Table S1 The search strategy of our review

**Database**	**Search strategy**	**Results**
**CNKI**	SU =结核病 AND FT='学校'+'高校'+ '中学'	**3753**
**Wanfang**	主题:(结核病*学校)	**1614**
**CBM**	("学校"[摘要]) AND "结核病"[摘要]	**891**
**PUBMED**	(tuberculosis[MeSH Major Topic]) AND outbreak[Title/Abstract] Filters: Free full text;	**304**
**Web of Science**	((TS=school) OR ((TS=university) OR (TS=college))) AND (TS= tuberculosis) AND (TS=outbreaks)	**134**
**EMBASE (ScienceDirect)**	TITLE-ABSTR-KEY(tuberculosis) and TITLE-ABSTR-KEY(school).	**331**

Science direct was the sub-database of EMBASE
