# Supplementary material for: Tuberculosis outbreaks among students in mainland China: a systematic review and meta-analysis
Source: BMC Infect Dis. 2019 Nov 14;19:972. doi: 10.1186/s12879-019-4573-3 (PMC6854678; doi:10.1186/s12879-019-4573-3)

Figure S1 Forest plot of total attack rates of different schools

Study ID	ES (95% CI)	% Weight
**Senior middle school**		
J Hao (2007)	0.04 (0.01, 0.10)	1.07
XQ Huang (2014)	0.01 (0.00, 0.02)	1.14
J Zhu (2013)	0.03 (0.01, 0.07)	1.13
TY Shen (2012)	0.02 (0.01, 0.04)	1.17
ZP Wang (2017)	0.00 (0.00, 0.01)	1.18
JS Song (2016)	0.01 (0.00, 0.02)	1.18
WJ Tang (2016)	0.02 (0.01, 0.05)	1.17
HW Hu (2012)	0.05 (0.02, 0.11)	1.17
H Xu (2016)	0.01 (0.01, 0.03)	1.18
YD Hong (2017)	0.03 (0.01, 0.06)	1.17
HZ Wu (2013)	0.02 (0.01, 0.04)	1.17
QD Ying (2018)	0.02 (0.01, 0.05)	1.17
CL Yin (2018)	0.02 (0.01, 0.03)	1.17
YF Shi (2015)	0.03 (0.01, 0.07)	1.20
XW Pang (2010)	0.01 (0.01, 0.03)	1.20
DH Li (2005)	0.00 (0.00, 0.01)	1.23
KY Luo (2006)	0.06 (0.03, 0.12)	1.24
GH Mao (2013)	0.01 (0.01, 0.03)	1.25
JM Ding (2002)	0.17 (0.09, 0.35)	1.22
SN Ding(1) (2014)	0.10 (0.05, 0.20)	1.23
B Wang (2014)	0.30 (0.15, 0.58)	1.24
YQ Hu (2011)	0.04 (0.02, 0.07)	1.27
YT Tian (2011)	0.04 (0.02, 0.07)	1.27
YJ He (2015)	0.02 (0.01, 0.04)	1.29
MS Chen (2009)	0.39 (0.20, 0.75)	1.24
QF Chen (2014)	0.03 (0.01, 0.05)	1.30
WY Jiang (2014)	0.22 (0.12, 0.41)	1.27
W Kong (2014)	0.01 (0.01, 0.02)	1.30
BC Lin (2007)	0.06 (0.03, 0.10)	1.30
DH Yu (2003)	0.07 (0.04, 0.12)	1.30
SJ Zhao (2013)	0.04 (0.03, 0.07)	1.31
XD Jiang (2006)	0.16 (0.09, 0.27)	1.30
CH Xue (2012)	0.03 (0.02, 0.05)	1.31
TX Qin (2012)	0.04 (0.02, 0.06)	1.31
XY Guo (2011)	0.13 (0.08, 0.21)	1.31
DB Hu (2011)	0.22 (0.13, 0.35)	1.32
J Zhu (2015)	0.20 (0.12, 0.31)	1.33
CQ Zhong (2008)	0.05 (0.04, 0.08)	1.34
XP Yang (2010)	0.03 (0.02, 0.05)	1.35
Yirong Fang et al (2013)	0.05 (0.03, 0.08)	1.35
ZX Chen (2010)	0.07 (0.05, 0.11)	1.35
W Yin (2009)	0.15 (0.10, 0.24)	1.34
M Ji (2007)	0.06 (0.04, 0.08)	1.36
QR Cui (2010)	0.31 (0.20, 0.47)	1.34
JY Chen (2013)	0.07 (0.05, 0.10)	1.36
XC Zhu (2011)	0.07 (0.05, 0.10)	1.37
XC Zhu (2012)	0.07 (0.05, 0.10)	1.37
LX Zheng (2010)	0.03 (0.02, 0.04)	1.38
Subtotal (I-squared = 91.9%, p = 0.000)	0.04 (0.03, 0.06)	60.53
.		
**University or college**		
C Ren (2015)	0.04 (0.02, 0.10)	1.13
SN Ding (2014)	0.08 (0.03, 0.17)	1.16
Y Wang (2014)	0.04 (0.02, 0.10)	1.17
XM Yang (2016)	0.14 (0.06, 0.32)	1.15
YH Wang (2009)	0.07 (0.03, 0.15)	1.16
J Tang (2017)	0.03 (0.01, 0.05)	1.20
LC Cui (2016)	0.01 (0.00, 0.02)	1.21
QQ Lv (2007)	0.08 (0.04, 0.18)	1.19
GX Fu (2010)	0.03 (0.01, 0.05)	1.25
QP Chen (2012)	0.25 (0.13, 0.48)	1.25
YB Wang (2016)	0.03 (0.01, 0.05)	1.27
YF Weng (2010)	0.15 (0.08, 0.28)	1.27
W Wang (2015)	0.04 (0.02, 0.07)	1.28
NZ Yang (2016)	0.08 (0.05, 0.14)	1.29
XT Yang (2016)	0.02 (0.01, 0.04)	1.30
XD Xu (2018)	0.03 (0.02, 0.05)	1.32
G Hu (2004)	0.01 (0.01, 0.02)	1.33
W Ji (2013)	0.11 (0.07, 0.18)	1.32
XP Zhao (2011)	0.02 (0.01, 0.02)	1.35
XX He (2014)	0.05 (0.03, 0.08)	1.34
LG Wang (2012)	0.27 (0.17, 0.43)	1.34
YY Fu (2012)	0.33 (0.21, 0.52)	1.34
XJ Xia (2013)	0.03 (0.02, 0.05)	1.36
CM Gao (2018)	0.04 (0.03, 0.05)	1.39
LM Wu (2006)	0.04 (0.03, 0.05)	1.39
DM Wang (2015)	0.02 (0.02, 0.03)	1.39
Subtotal (I-squared = 92.3%, p = 0.000)	0.05 (0.04, 0.07)	33.16
.		
**Junior middle school**		
G Huang (2013)	0.05 (0.02, 0.09)	1.24
Y Qi (2014)	0.12 (0.06, 0.24)	1.25
Jun L (2017)	0.14 (0.07, 0.28)	1.25
J Zheng (2016)	0.04 (0.02, 0.07)	1.27
LR Peng (2012)	0.02 (0.01, 0.04)	1.30
Subtotal (I-squared = 85.4%, p = 0.000)	0.06 (0.03, 0.12)	6.31
.		
Overall (I-squared = 91.7%, p = 0.000)	0.05 (0.04, 0.06)	100.00

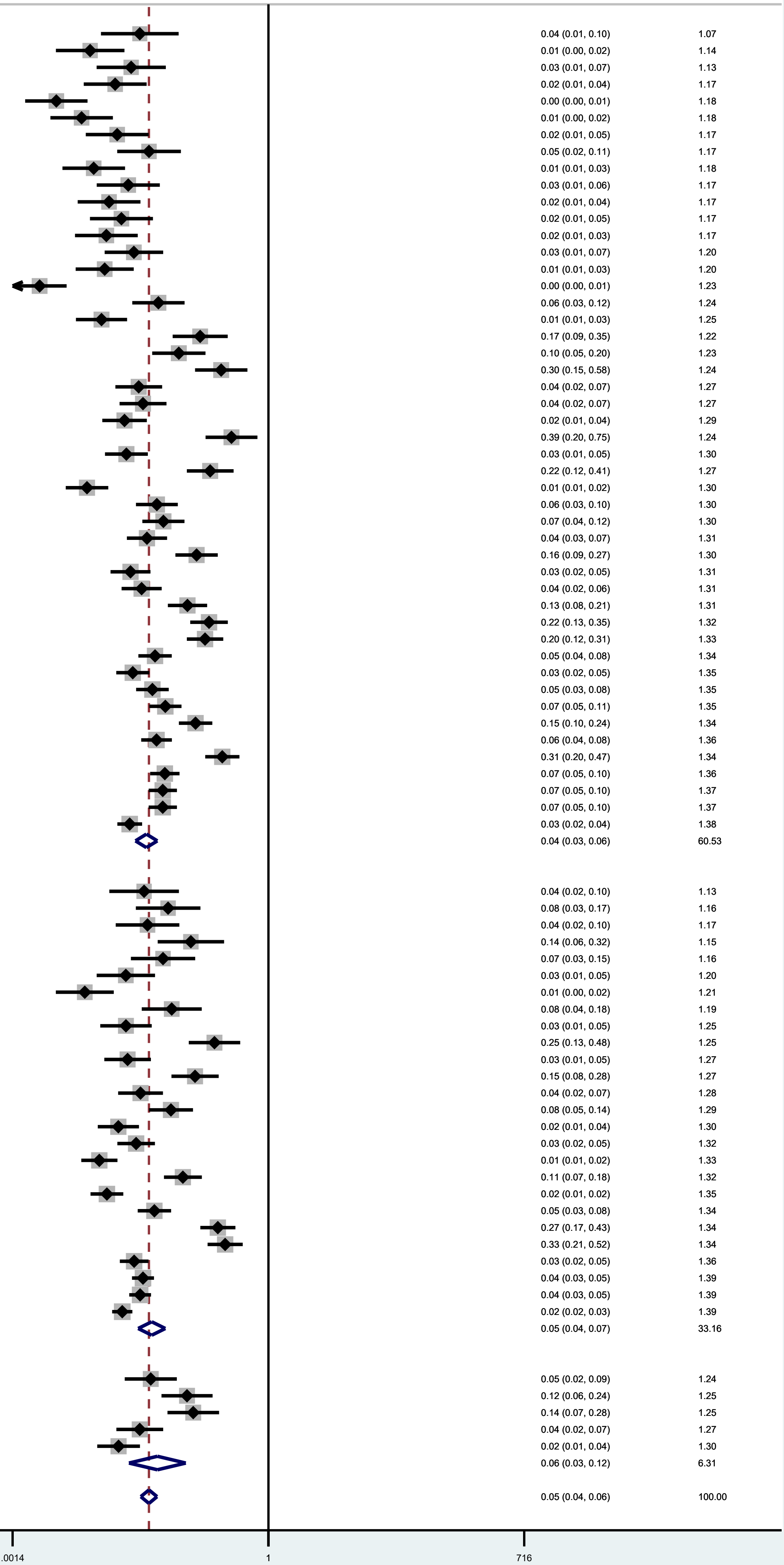

NOTE: Weights are from random effects analysis

Figure S2 Forest plot of total attack rates of different regions where the outbreaks schools located

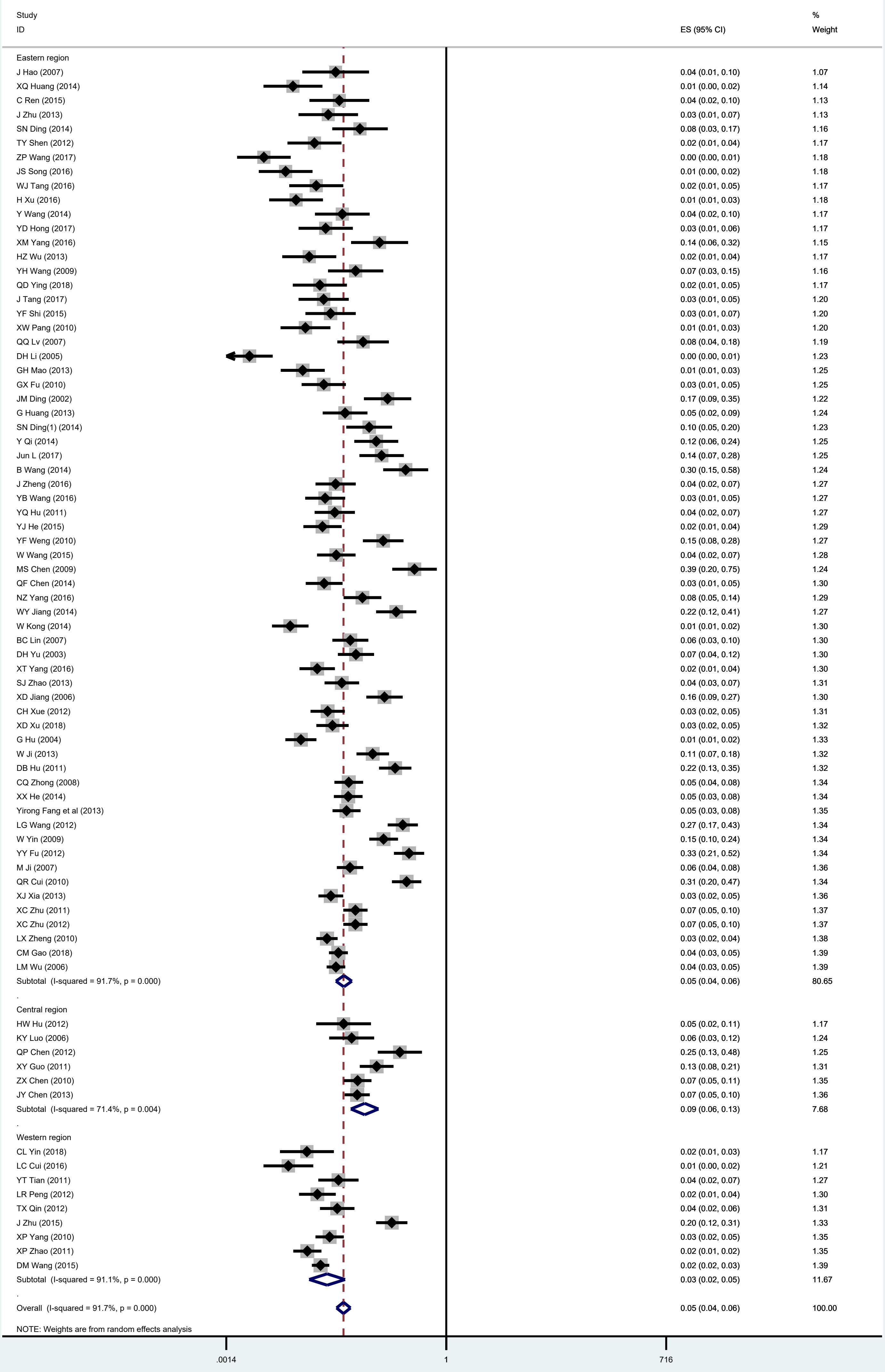

Figure S3 Forest plot of total attack rates of different case number

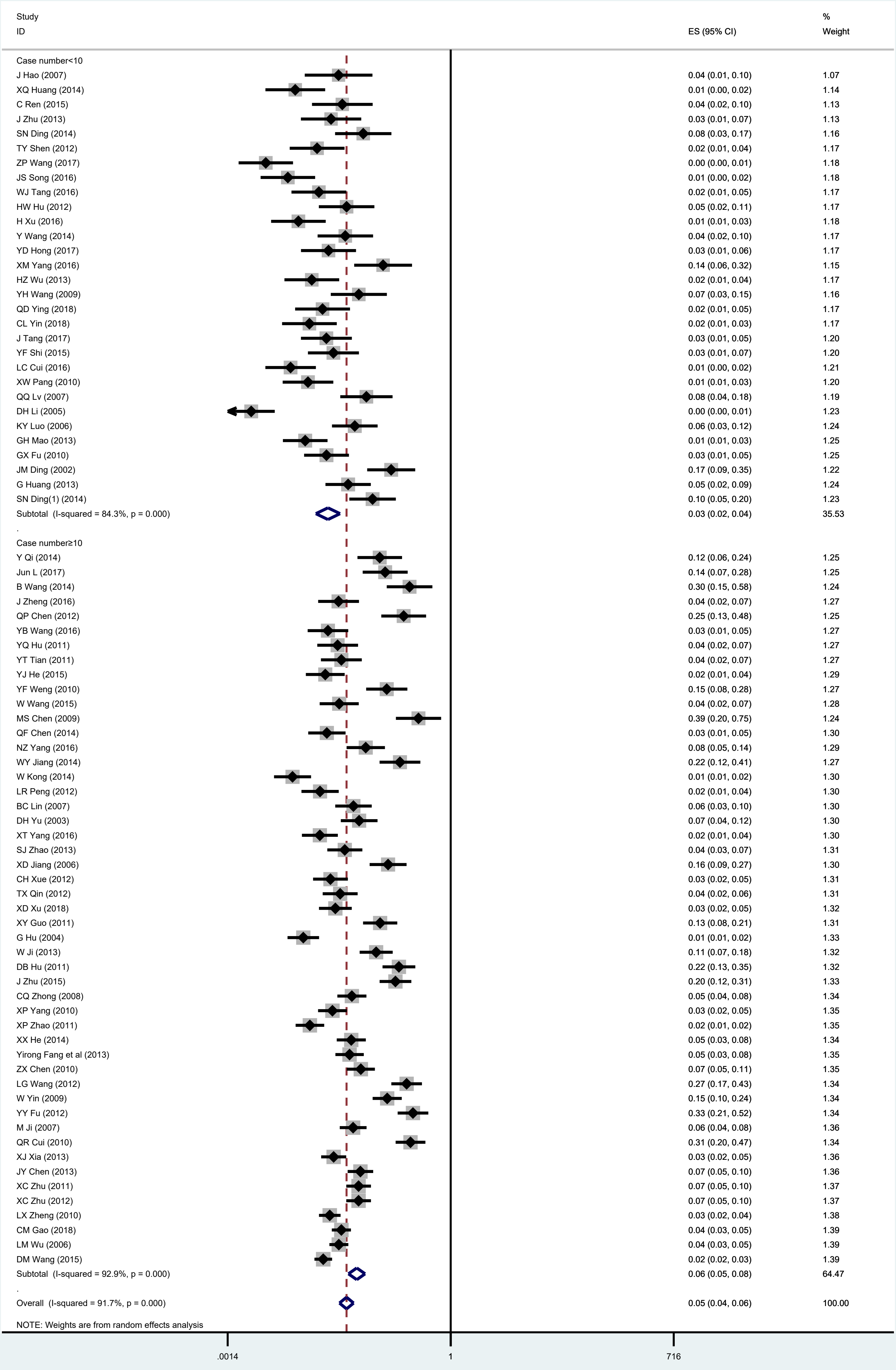

Supplement: Supplementary file 2 — Additional file 2: Figure S1. Forest plot of total attack rates of different schools, Figure S2. Forest plot of total attack rates of different regions where the outbreaks schools located and Figure S3. Forest plot of total attack rates of different case number. [file 12879_2019_4573_MOESM2_ESM.pdf]
