# Supplementary material for: Tuberculosis outbreaks among students in mainland China: a systematic review and meta-analysis
Source: BMC Infect Dis. 2019 Nov 14;19:972. doi: 10.1186/s12879-019-4573-3 (PMC6854678; doi:10.1186/s12879-019-4573-3)

Figure S4 Forest plot of class attack rates of different schools

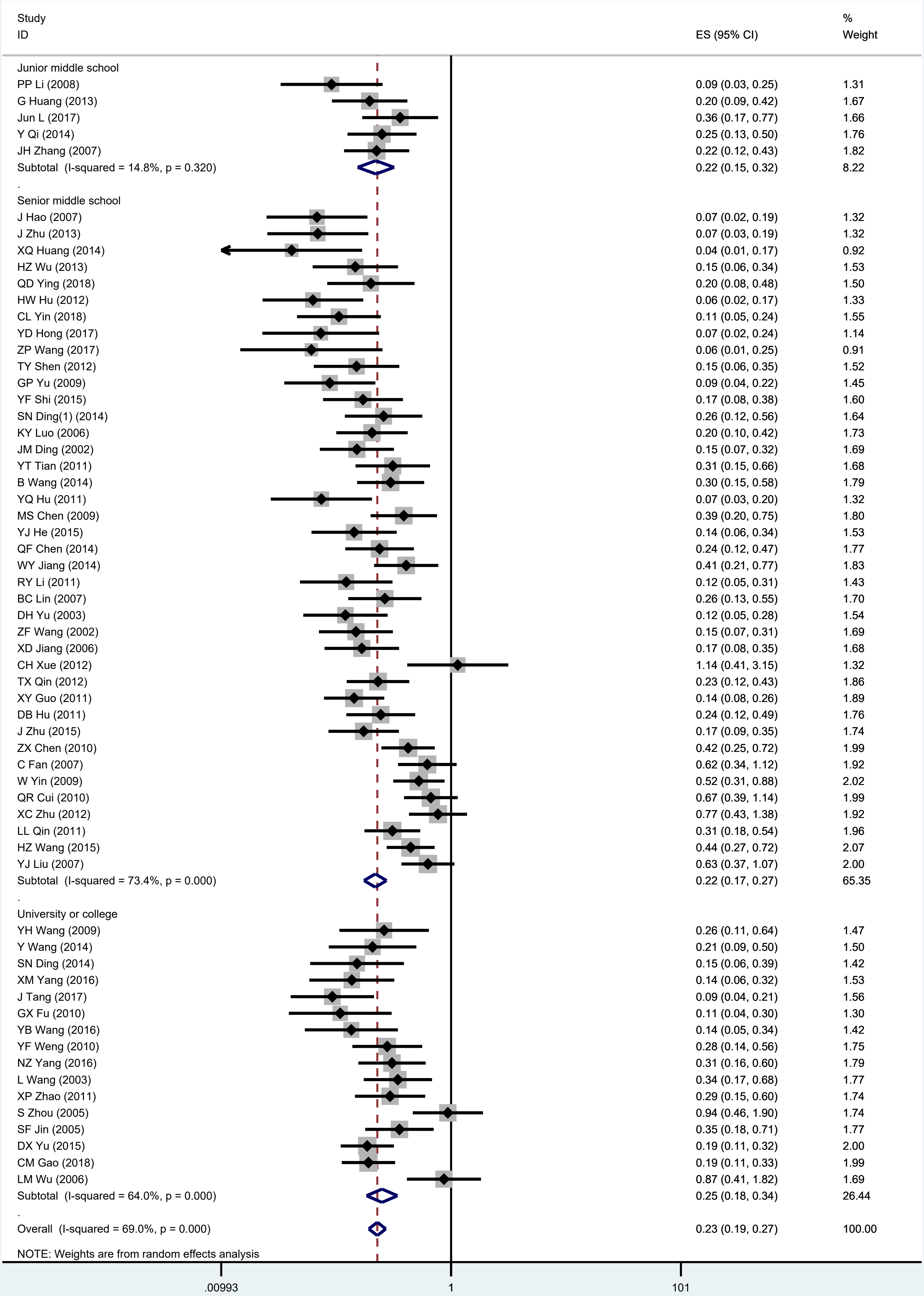

Figure S5 Forest plot of class attack rates of different regions where the outbreaks schools located

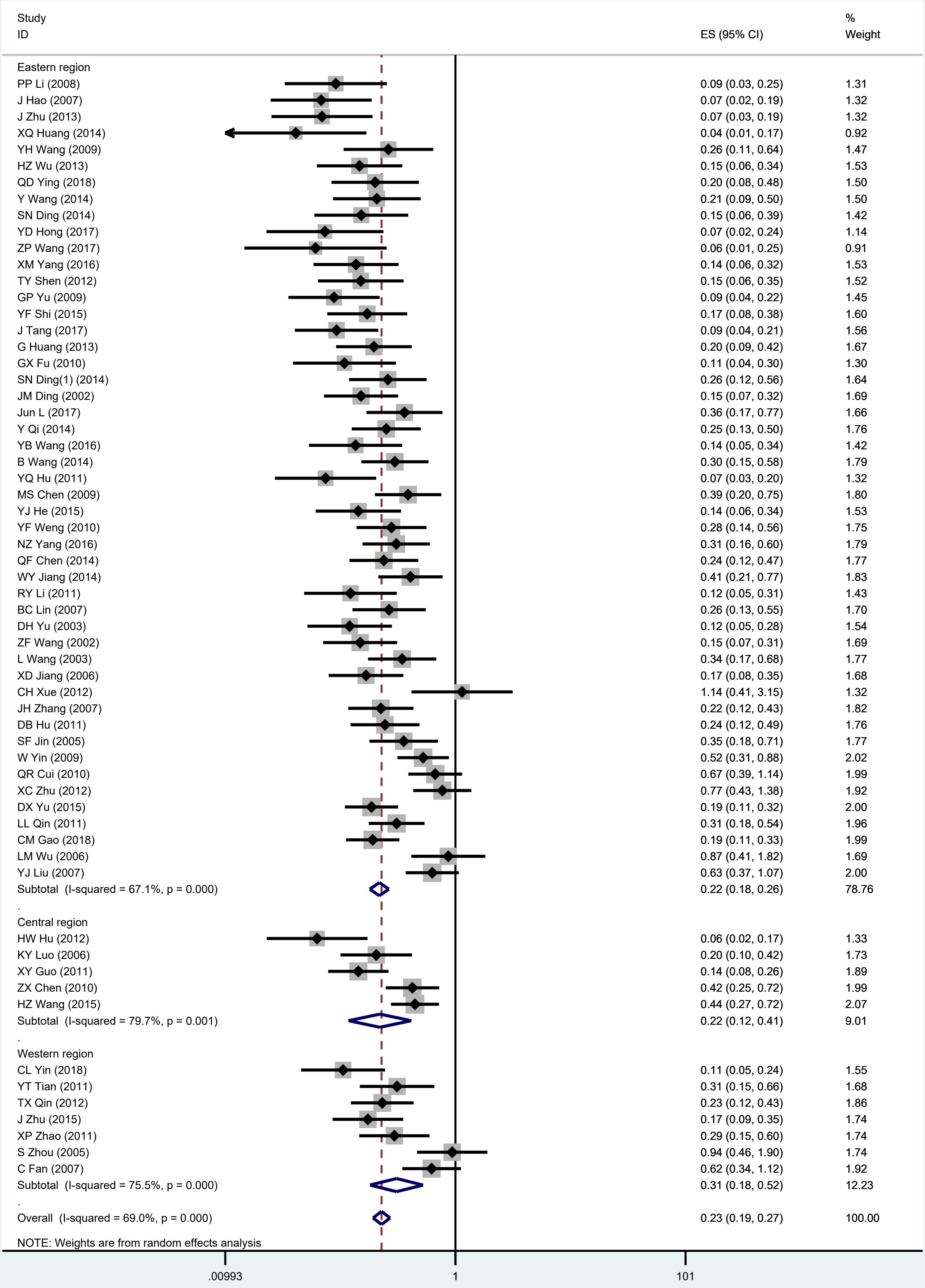

Figure S6 Forest plot of class attack rates of different case number

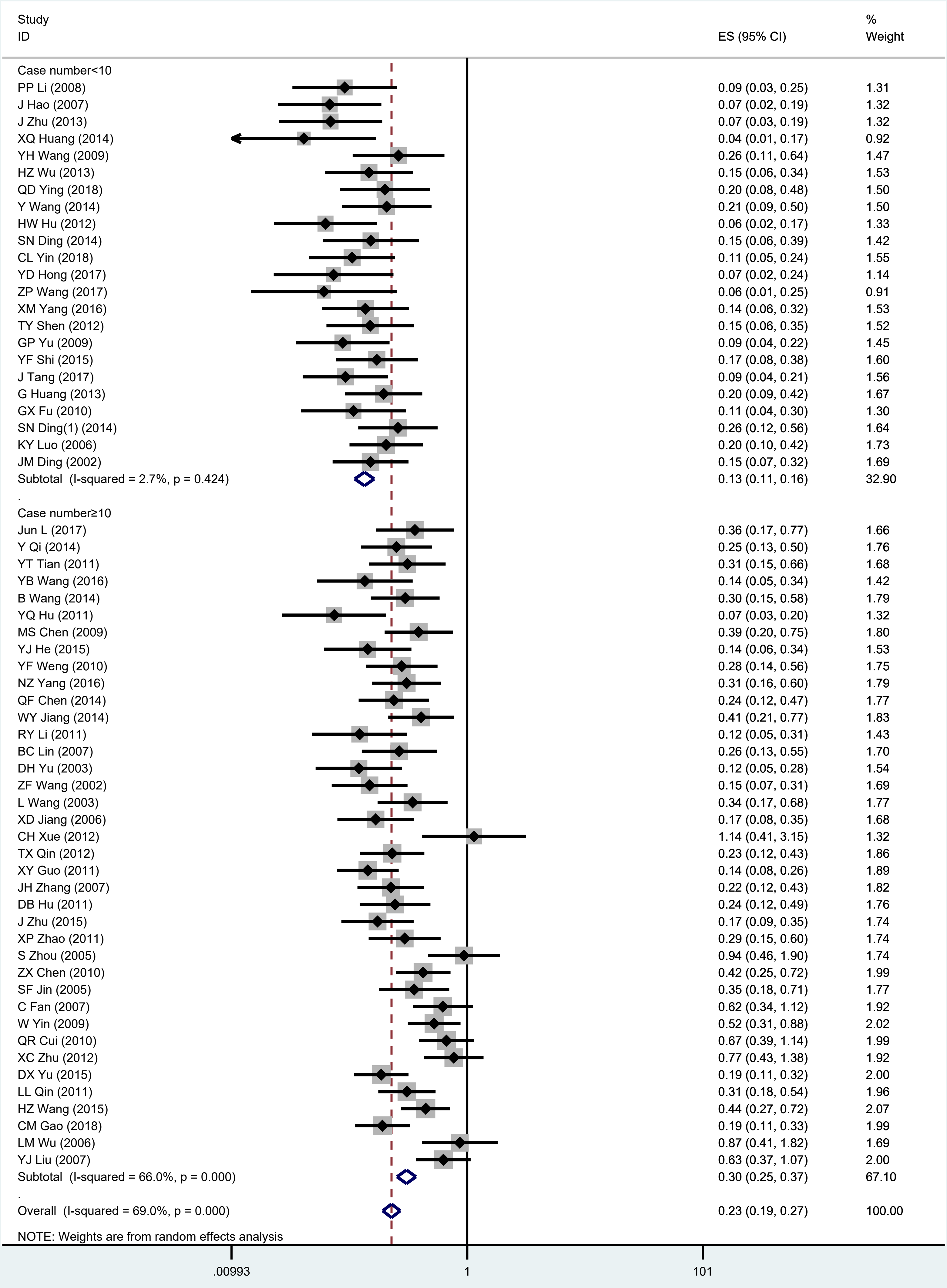

Figure S7 Forest plot of class attack rates of different diagnose interval of index cases

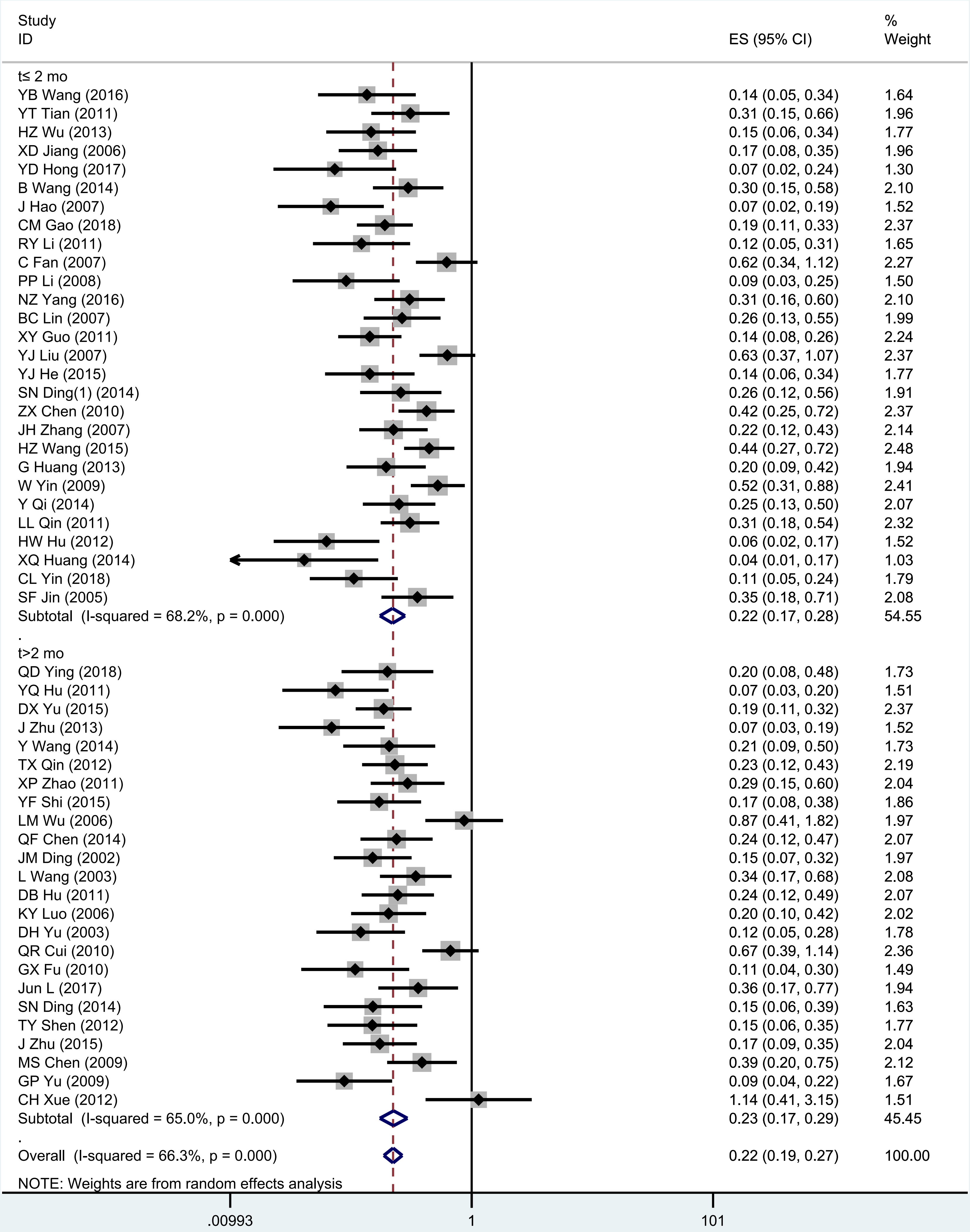

Supplement: Supplementary file 3 — Additional file 3: Figure S4. Forest plot of class attack rates of different schools, Figure S5. Forest plot of class attack rates of different regions where the outbreaks schools located, Figure S6. Forest plot of class attack rates of different case number, Figure S7. Forest plot of class attack rates of different diagnose interval of index cases. [file 12879_2019_4573_MOESM3_ESM.pdf]
