# Supplementary material for: Tuberculosis outbreaks among students in mainland China: a systematic review and meta-analysis
Source: BMC Infect Dis. 2019 Nov 14;19:972. doi: 10.1186/s12879-019-4573-3 (PMC6854678; doi:10.1186/s12879-019-4573-3)
Supplement: Supplementary file 4 — Additional file 4: Table S2. The outcomes of each outbreak for the meta-analysis. [file 12879_2019_4573_MOESM4_ESM.pdf]

Table S2 The outcomes of each outbreak for the meta-analysis

Author	Year	The No. of students in class	The No. of patients in class	The screened population	The LTBI cases	Patients
Y Qi	2014	50	10	91	61	10
MS Chen	2009	43	12	43	22	12
JM Ding	2002	61	8	61	21	9
XM Yang	2012	50	6	50	36	6
CH Xue	2012	15	8	534	134	15
GH Mao	2013			665	56	9
XJ Xia	2013			942		29
YT Tian	2011	38	9	287		11
DH Yu	2003	56	6	222	36	14
G Hu	2004			1405	178	18
DH Li	2005			2869	112	8
XD Jiang	2006	56	8	110	41	15
KY Luo	2006	53	9	161	30	9
LM Wu	2006	28	13	1429	189	51
J Hao	2007	63	4	113	19	4
BC Lin	2007	43	9	260	13	14
QQ Lv	2007			91	37	7
CQ Zhong	2008			427	104	22
YH Wang	2009	29	6	96	31	6
W Yin	2009	64	22	180	24	24
ZX Cheng	2010	64	19	363	36	24
QR Cui	2010	55	22	111	42	26
GX Fu	2010	41	4	359	89	9
YF Weng	2010	46	10	91	22	12
XP Yang	2010			740	135	22
LX Zheng	2010			1416	15	39
XY Guo	2011	96	12	153	31	17
DB Hu	2011	51	10	112	14	20
YQ Hu	2011	58	4	319	89	11
XP Zhao	2011	44	10	1418	105	22
QP Chen	2012			55	27	11
YY Fu	2012			101	94	25
HW Hu	2012	68	4	135	24	6
LR Peng	2012			624	18	13
TY Shen	2012	46	6	314	33	6
LG Wang	2012			112	23	24
XC Zhu	2012	46	20	499	136	31
JY Chen	2013			444	142	29
G Huang	2013	49	8	194	75	9

W Ji	2013			180	85	18
HZ Wu	2013	47	6	366	73	6
SJ Zhao	2013			355	76	15
J Zhu	2013	62	4	175	25	5
QF Chen	2014	52	10	513	94	13
SN Ding(1)	2014	39	8	99	44	9
SN Ding	2014	38	5	85	39	6
XQ Huang	2014	51	2	493	6	5
WY Jiang	2014	45	13	71	30	13
W Kong	2014			1389	40	13
B Wang	2014	48	11	48	7	11
Y Wang	2014	35	6	140	78	6
YJ He	2015	48	6	496	75	12
C Ren	2015			127	11	5
YF Shi	2015	48	7	229	31	7
DM Wang	2015			2539		58
W Wang	2015			333	128	12
J Zhu	2015	61	9	128		21
LC Cui	2016			791	78	7
JS Song	2016			735	39	6
WJ Tang	2016			298	92	6
YB Wang	2016	42	5	421	41	11
H Xu	2016			540	69	6
NZ Yang	2016	47	11	172	34	13
XT Yang	2016			676		14
J Zheng	2016			311	73	11
YD Hong	2017	44	3	226		6
Yirong	2013			476	122	23
Fang et al						
M Ji	2007			487	158	26
XW Pang	2010			477	95	7
XC Zhu	2011			499	113	31
TX Qin	2012	64	12	405		15
XX He	2014			434	203	22
ZF Wang	2002	62	8	728	140	14
L Wang	2003	43	11	900	224	15
C Fan	2007	47	18	1083		24
SY Guo	2014			859	63	8
BS He	2009			3193	591	51
QG Xie	2012			3511	132	12
SF Jin	2005	42	11	1533	95	24
AH Li	2004			1659	110	27
JC Li	2012			3524	307	10
PP Li	2008	48	4	1623	58	4

RY Li	2011	46	5	2648	393	14
YJ Liu	2007	57	22	2516	263	66
YN Piao	2008			699	149	27
LL Qin	2011	68	16	2423	125	37
QZ Wang	2012			719	106	14
HZ Wang	2015	78	24	4041	142	42
ML Wang	2006			2011	267	18
WB Wang	2005			5053		14
K Xu	2015			816	72	8
SB Yang	2004			1344		13
YC Yang	2008			693		8
DX Yu	2016	102	16	587	56	31
GP Yu	2009	62	5	1116	108	6
XM Zhang	2007			3790	374	25
JH Zhang	2007	60	11	2506	272	19
DY Zhao	2013			1336	65	23
RX Zhao	2013			2053	294	28
S Zhou	2005	31	15	2588		23
Jun L	2017	34	9	79	18	10
J Tang	2017	71	6	280	34	7
ZP Wang	2017	35	2	1402	201	6
CM Gao	2018	100	16	1305	412	50
XD Xu	2018		11	526	152	17
CL Yin	2018	63	6	392	4	6
QD Ying	2018	36	6	268	22	6

1. Qi Y, Lu HY, Yang LJ, Yang YT, Zhou Y, Lu XW: **The retrospective analysis of a tuberculosis outbreak in a middle school**. *Journal of Tuberculosis and Lung Health* 2014, **3**(3):161-165. (In Chinese)
2. Chen MS, Jin CQ, Shao JP: **Epidemiological investigation of an outbreak of tuberculosis in a school**. *China Preventive Medicine* 2009, **10**(07):675-676. (In Chinese)
3. Ding JM, Han WC, Zhang J, Pang HB: **A survey and disposal of an outbreak of pulmonary tuberculosis among students**. *Occupation and Health* 2002, **18**(11):71-72. (In Chinese)
4. Yang XM, Zhang SS: **Epidemiological analysis of an outbreak of tuberculosis among college students**. *Journal of Medical Pest Control* 2012, **28**(10): 1168, 1171. (In Chinese)
5. Xue CH, Sun BB: **An investigation and analysis of tuberculosis outbreak in middle School students**. *Chinese Journal of School Doctor* 2012, **26**(05):370-371. (In Chinese)
6. Mao GH, Yu M, Lin X: **Epidemiological investigation of a tuberculosis outbreak in a middle school in Ningbo City**. *Modern Practical Medicine* 2013, **25**(05):566-567. (In Chinese)
7. Xia XJ, Jiang H, Luo WH, Dai B: **Analysis of an outbreak of tuberculosis in a university in Jiangsu**. *Modern Preventive Medicine* 2013, **40**(21):4082-4084. (In Chinese)
8. Tian YT, Li Z, Xiong XH: **The investigation and disposal of tuberculosis outbreak in a senior high school student in Yubei District**. *Practical Preventive Medicine* 2011, **18**(10):1886-1887. (In Chinese)
9. Yu DH, Du CN, Li Y: **Investigation of an outbreak of tuberculosis in a middle school in Weihai City**. *Literature and Information of Preventive Medicine* 2003, **9**(05):554. (In Chinese)
10. Hu G, Zhang JX, Jiang DM: **An investigation on collective infection of tuberculosis in a university**. *Occupation and Health* 2004, **20**(07):80. (In Chinese)
11. Li DH, Liu T: **Investigation and analysis of an outbreak of pulmonary tuberculosis in a rural middle school**. *Chinese Primary Health Care* 2005, **19**(12):57. (In Chinese)
12. Jiang XD: **An investigation of tuberculosis outbreak among senior middle school students in Zhejiang Province**. *Chinese Journal of School Health* 2006, **27**(05):453. (In Chinese)
13. Luo KY, Liao SJ, Wang XF, Li HH, Jiang LN, Gui B, Zhang HY, Li J, Cheng DJ: **Analysis of an outbreak of tuberculosis in schools**. *Practical Preventive Medicine* 2006, **13**(04):939-940.

(In Chinese)

14. Wu LM, Luo J, Lu M, Wang L, Hu ZQ, Wang M: **Analysis of an outbreak of TB in an university in Hangzhou**. *Journal of Medical Research* 2006, **35**(06):61-63. (In Chinese)

15. Hao J, Tan HM, Xie YZ, Zhang M: **Investigation on the prevalence of tuberculosis among students in a secondary school in Pingyin County in 2006**. *Preventive Medicine Tribune* 2007, **13**(05):476. (In Chinese)

16. Lin BC, Huang SH: **An investigation of tuberculosis outbreaks among high school students**. *Occupation and Health* 2007, **23**(14):1231-1232. (In Chinese)

17. Lv QQ, Ju YX, Li YY: **Investigation of an outbreak of tuberculosis among college students**. *Chinese Journal of School Health* 2007, **28**(12):1147. (In Chinese)

18. Zhong CQ: **An on-campus tuberculosis outbreak investigation and disposal**. *Chinese Journal of Pest Control* 2008, **24**(08):604-605. (In Chinese)

19. Wang YH: **Analysis of an outbreak of tuberculosis in a certain university**. *Chinese Journal of School Doctor* 2009, **23**(05):540-541. (In Chinese)

20. Yin W, Xue H, Zhang CG: **Analysis of an outbreak of tuberculosis in a certain university**. *Jiangsu Journal of Preventive Medicine* 2009, **20**(02):45-46. (In Chinese)

21. Cheng ZX, Hu LJ, Fang YR, Li YX, Wang R: **Epidemiologic survey on tuberculosis outbreak which happened at a high school in Wuhu**. *World Journal of Infection* 2010, **10**(4):195-198. (In Chinese)

22. Cui QR, Ge JH, Pang MW, Chen YL, Xie WF: **Survey of a school cluster of pulmonary tuberculosis**. *Disease Surveillance* 2010, **25**(04):335-337. (In Chinese)

23. Fu GX, Shao YX, Chen H: **Epidemiological investigation of an outbreak of tuberculosis among college students**. *Chinese Journal of School Health* 2010, **31**(02):244-245. (In Chinese)

24. Weng YF, Zhang JY: **Survey of a tuberculosis epidemic in a university in Zhoushan city, Zhejiang province**. *Disease Surveillance* 2010, **25**(10):842-843. (In Chinese)

25. Yang XP, Yang SJ: **Analysis of an outbreak of tuberculosis in a school**. *Chinese Journal of School Health* 2010, **31**(09):1144. (In Chinese)

26. Zheng LX, Long HP, Zheng SJ, Xiao ML, Li B: **Epidemiological investigation after tuberculosis outbreak in a middle school**. *Practical Preventive Medicine* 2010,

17(04):672-673. (In Chinese)

27. Guo XY, Shang XS, Fan J: **Investigation and analysis of a tuberculosis outbreak**. *Journal of Public Health and Preventive Medicine* 2011, **22**(05):98-99. (In Chinese)

28. Hu DB, Wang B: **Epidemiological investigation of outbreaks of tuberculosis in schools**. *Shanghai Journal of Preventive Medicine* 2011, **23**(02):54-55. (In Chinese)

29. Hu YQ, Shen SQ, Jiang W: **A school tuberculosis cluster epidemic survey**. *Zhejiang Journal of Preventive Medicine* 2011, **23**(05):43-44. (In Chinese)

30. Zhao XP, Zhang TH: **Investigation and disposal for clustered tuberculosis cases in a professional technology college**. *Occupation and Health* 2011, **27**(24):2912-2913. (In Chinese)

31. Chen QP, Chen TM, Zhao HW: **Assessment on investigation and control measures of tuberculosis outbreaks in college**. *Chinese Journal of School Health* 2012, **33**(02):172-173,176. (In Chinese)

32. Fu YY, Zhang GQ, Wei WL, Zhang YH, Shang J, Zhong D: **Epidemiological analysis of tuberculosis outbreak among internship students in a vocational and technical college**. *Chinese Journal of School Health* 2012, **33**(05):621-622. (In Chinese)

33. Hu HW, Liu ML: **Investigation report on pulmonary tuberculosis aggregation on campus**. *Anhui Journal of Preventive Medicine* 2012, **18**(04):299-300. (In Chinese)

34. Peng LR, Zhang L, Peng R: **Tuberculosis outbreak in Kangding middle school**. *China Practical Medical* 2012, **7**(26):250-251. (In Chinese)

35. Shen TY, Li JY, Fu LJ: **Investigation and analysis of a clustered pulmonary tuberculosis case in a school in shaoxing county**. *Chinese Rural Health Service Administration* 2012, **32**(09):937-939. (In Chinese)

36. Wang LG, Zhu JW: **Investigation of a tuberculosis outbreak in a school**. *Zhejiang Journal of Preventive Medicine* 2012, **24**(05):46-47. (In Chinese)

37. Zhu XC, Ying XJ, Wang Y, Weng LX, Qiu L: **Investigation of pulmonary tuberculosis outbreak in a school**. *Chinese Journal of School Health* 2012, **33**(04):453-454. (In Chinese)

38. Chen JY, Shi SJ, Fang XH, Pan Y: **Report on the investigation and disposal of a tuberculosis outbreak in a middle school in Jing County**. *Anhui Journal of Preventive Medicine* 2013, **19**(01):35-36, 67. (In Chinese)

39. Huang G, Zhou XT, Zhang CM: **Field epidemiological investigation on TB outbreak in a middle school of Shaoxing City**. *Occupation and Health* 2013, **29**(02):124, 257. (In Chinese)

40. Ji W: **Investigation of tuberculosis outbreaks in Tibetan classes in a child care school**. *Jiangsu Health Care* 2013, **15**(04):7-8. (In Chinese)

41. Wu HZ, Hu YQ: **An investigation of tuberculosis cluster infection in high school students.** *Zhejiang Journal of Preventive Medicine* 2013, **25**(04):46-47, 50. (In Chinese)

42. Zhao SJ, Zhang YQ, Guo JH: **Investigation and analysis of an outbreak of tuberculosis in a school**. *Shanxi Medical Journal* 2013, **42**(12):1368-1369. (In Chinese)

43. Zhu J, Fan FN, Xu JY: I**nvestigation on the outbreak of tuberculosis aggregation in senior three students**. *Chinese Journal of School Health* 2013, **34**(06):748-749. (In Chinese)

44. Chen QF, Gao HQ: **Investigation on tuberculosis aggregation in a middle School**. *Zhejiang Journal of Preventive Medicine* 2014, **26** (08):824-826. (In Chinese)

45. Ding SN, Sun ZP, Li C, Nie JQ, Liu R, Liu X, Ji W: **Epidemiologic survey on an outbreak of tuberculosis in a school in Jiangsu**. *Modern Preventive Medicine* 2014, **41**(07):1164-1166, 1169. (In Chinese)

46. Ding SN, Sun ZP, Li C, Nie JQ, Liu R, Liu X, Zang TY: **Epidemiologic survey of an outbreak tuberculosis in a university in Nanjing City**. *Chinese Journal of School Doctor* 2014, **28**(02):124-126,128. (In Chinese)

47. Huang XQ, WU WY, Huang LM: **Tuberculosis aggregation report in middle schools in a mountainous area**. *Zhejiang Journal of Preventive Medicine* 2014, **26**(11):1143-1144. (In Chinese)

48. Jiang WY, Shao JP, Lin BC: **Investigation on tuberculosis aggregation in high school student in Wenling City**. *Chinese Journal of School Health* 2014, **35**(07):1097-1099. (In Chinese)

49. Kong W, Ding XY, Lu W, Liu Q: **Investigation on pulmonary tuberculosis in a middle school in Jiangsu Province**. *Jiangsu Journal of Preventive Medicine* 2014, **25**(04):54-55. (In Chinese)

50. Wang B, Wang ZG, Gu MX, Zheng JJ, Yu YJ: **Epidemiological investigation of tuberculosis aggregation in a school**. *Chinese Journal of School Health* 2014, **35**(08):1253-1254. (In Chinese)

51. Wang Y, Yang SQ, Luan H, Liu XY: **Investigation and analysis of pulmonary tuberculosis epidemics among college students**. *Chinese Journal of Health Laboratory Technology* 2014, **24**(03):432-434. (In Chinese)

52. He YJ, Huang MP, Cao Y, Liang ZQ: **Analysis of a pulmonary tuberculosis outbreak and follow-up of 3 years in a school**. *The Journal of Medical Theory and Practice* 2015, **28**(13):1810-1811. (In Chinese)

53. Ren C, Gao L, Huang SY, Deng YR, Ying LP: I**nvestigation and analysis of tuberculosis aggregation in a school**. *Modern Practical Medicine* 2015, **27**(08):1025-1026. (In Chinese)

54. Shi YF, Miao C, Shen XL: **Investigation report on tuberculosis aggregation in a vocational middle school**. *Zhejiang Journal of Preventive Medicine* 2015, **27**(02):164-166. (In Chinese)

55. Wang DM, Wang JX, Wang XL: **Analysis of tuberculosis aggregation in a university**. *Chinese Journal of Coal Industry Medicine* 2015, **18**(12):2086-2089. (In Chinese)

56. Wang W, Zhou W, Lou MJ, Chen HJ: **Investigation on tuberculosis aggregation in a university in Ningbo city**. *Shanghai Journal of Preventive Medicine* 2015, **27**(11):702-703,708. (In Chinese)

57. Zhu J, Gu YX, Zhang YG: **An investigation report on an outbreak of tuberculosis in schools**. *The Medical Forum* 2015, **19**(07):995-997. (In Chinese)

58. Cui LC, Mu PH: **Investigation and analysis of tuberculosis aggregation in colleges and universities**. *Chinese Journal of Antituberculosis* 2016, **38**(1):74-76. (In Chinese)

59. Song JS, Liu YJ: **A school TB epidemiological investigation and disposal**. *WorldLatest Medicine Information* 2016, **16**(60):183,188. (In Chinese)

60. Tang WJ, Yang WQ, He FH, Feng DZ: **Investigation on tuberculosis aggregation in a middle school in Jinhu County**. *Jiangsu Journal of Preventive Medicine* 2016, **27**(03):317-318. (In Chinese)

61. Wang YB, Zhao X: **An epidemiological investigation of tuberculosis aggregation in a school in Xinle City**. *Journal of Medical Pest Control* 2016, **32**(12):1413-1414. (In Chinese)

62. Xu H, Sun B: **Report on a pulmonary tuberculosis cluster epidemic occurred in a senior high school, Donghai County**. *Modern Preventive Medicine* 2016, **43**(17):3217-3220. (In Chinese)

63. Yang NZ, Chen Y, Yang WY, Wang SS, Ni Z: **An analysis of tuberculosis clustered**

outbreaks in schools. *Preventive medicine* 2016, **28**(11):1146-1149. (In Chinese)

64. Yang XT, Xiang LH, Liu XF, Wang N, Ma Y, Hu WH, Cao M: **An epidemiological investigation of a tuberculosis outbreak in a university in Shanghai**. *Chinese Journal of School Health* 2016, **37**(12):1902-1904. (In Chinese)

65. Zheng J: **Investigation and interventions on an outbreak of tuberculosis**. *China Tropical Medicine* 2016, **16**(02):184-185, 189. (In Chinese)

66. Hong YD, Xue FH, Chen Q: **Investigation on the pulmonary tuberculosis epidemics in a vocational school**. *Chinese Rural Health Service Administration* 2017, **37**(02):169-171. (In Chinese)

67. Fang Y, Zhang L, Tu C, Ye D, Fontaine R, Ma H, Hao J, Fu L, Ying X, Chen Q *et al*: **Outbreak of pulmonary tuberculosis in a Chinese high school, 2009-2010**. *J Epidemiology* 2013, **23**(4):307-312.

68. Ji M, Chen J, Zhu W, Zhou Y: **Investigation and analysis of a tuberculosis epidemic in middle school**. *Chinese Journal of Antituberculosis* 2013, **35**(11):941-943. (In Chinese)

69. Pang XW, Zhang D, Fu YY: **Analysis of screening results of close contacts after tuberculosis outbreak in a school**. *Chinese Journal of School Health* 2015, **36**(8):1265-1267. (In Chinese)

70. Zhu XC, Ying XJ, Wang Y, Weng LX, Qiu L: **Investigation and disposal of pulmonary tuberculosis aggregation in a school**. *The 19th Zhejiang Rural Health Reform and Development Conference; Shaoxing, Zhejiang, China*; 2011: 5. (In Chinese)

71. Qin TX, Yin N, Zhao RX: **Analysis of tuberculosis aggregation in a school**. *Chinese Journal of School Doctor* 2015, **29**(03):193-194. (In Chinese)

72. He XX, Luo P, Li B, Gao ZD: **Surveillance and disposal of tuberculosis in a certain university in Beijing**. *Chinese Journal of Antituberculosis* 2014, **36**(12):1101-1104. (In Chinese)

73. Wang ZF: **Investigation of tuberculosis outbreak in a middle school in Rongcheng City**. *Literature and Information of Preventive Medicine* 2002, **8**(01):26. (In Chinese)

74. Wang L, Wu LM, Lu M, Hu ZQ, Zhang YZ: **An Investigation of outbreak of tuberculosis among students and countermeasures**. *Chinese Journal of School Health* 2003, **24**(05):539-540. (In Chinese)

75. Fan C, Hu DY, Zhou KX, Su Q: **Analysis of 24 cases of tuberculosis in a third grade in a middle school in Chongqing**. *Chongqing Medicine* 2007, **36**(19):2004-2005. (In Chinese)

76. Guo SY, Liang MH, Qiao HY, Zheng FF: **A clustered tuberculosis epidemic investigation report**. *Henan Journal of Preventive Medicine* 2014, **25**(04):302-303, 316. (In Chinese)

77. He BS, Dai XP, Xiang QH, Li SC, Chen H, Wang J, Hu XQ, Guo YX, Yu HH: **Report on the investigation and disposal of tuberculosis outbreak in the first middle school in Xiaogan City**. *Chinese Journal of Antituberculosis* 2009, **31**(11):668-671. (In Chinese)

78. Xie QG, Li ZX: **Epidemiological survey of tuberculosis among middle school students**. *Medical Innovation of China* 2012, **9**(10):102-103. (In Chinese)

79. Jin SF: **An analysis of tuberculosis epidemic in colleges and universities**. *Dandong Medicine* 2005, (3):37. (In Chinese)

80. Li AH: **An investigation report of a tuberculosis outbreak**. *Occupation and Health* 2004, **20**(06):88. (In Chinese)

81. Li JC: **Analysis of tuberculosis outbreak in schools**. *Chinese Community Doctors* 2012, **14**(16):399-400. (In Chinese)

82. Li PP, Zhang HL, Wan CG: **Investigation and handling of the epidemic situation of tuberculosis in a middle school in Yandu District of Yancheng City**. *Occupation and Health* 2008, **24**(07):672-673. (In Chinese)

83. Li RY, Wang SP, Yu JP: **Epidemiological survey of tuberculosis clusters in a school in Weihai City**. *Preventive Medicine Tribune* 2011, **17**(12):1121-1123. (In Chinese)

84. Liu YJ, Liu CS, Wang GY, Li HB, Pan P, Nie JS, Yang YL: **Epidemic investigation and disposal of tuberculosis outbreak in a high school in Chaoyang City, Liaoning Province**. *Journal of Tropical Diseases and Parasitology* 2007, **5**(1):49-51. (In Chinese)

85. Piao YN, Jin H: **Disposal and analysis of tuberculosis outbreak in a high school in Hunchun**. *The Journal of the Chinese Antituberculosis Association* 2008, **30**(02):149-150. (In Chinese)

86. Qin LL: **Investigation and analysis of tuberculosis outbreak in a high school**. *Chinese Journal of School Doctor* 2011, **25**(7):526,528. (In Chinese)

87. Wang QZ, Fang XH: **Site investigation and analysis of a tuberculosis outbreak in a high school**. *Journal of Anhui Health Vocational & Technical college* 2012, **11**(04):101-102, 108.

(In Chinese)

88. Wang HZ, Chang JH, Li PJ: **An epidemiological investigation of tuberculosis outbreaks in schools**. *Chinese Journal of School Health* 2015, **36**(04):618-619. (In Chinese)

89. Wang ML, Li XP, Zhao W, Wang FY: **School tuberculosis epidemiological investigation and interventions.** *Journal of Qiannan Medical College for Nationalities* 2006, **19**(1):37-38. (In Chinese)

90. Wang WB: **Investigation of tuberculosis outbreak in a high school student in Gansu Province**. *Chinese Journal of School Health* 2005, **26**(11):74. (In Chinese)

91. Xu K, Zheng LL: **Epidemiological analysis of an outbreak of tuberculosis in a high school**. *China Modern Doctor* 2015, **53**(05):118-120. (In Chinese)

92. Yang SB, Li Q, Miao ZP: **An outbreak of tuberculosis among school students**. *Chinese journal of school health* 2004, **25**(06):750. (In Chinese)

93. Yang YC: **Survey on tuberculosis outbreak at a boarding school in Hongyuan County**. *Journal of preventive medicine* 2008, **24**(09):742-743. (In Chinese)

94. Yu DX, liu JW: **Epidemiological survey on a tuberculosis outbreak in a military academy**. *Modern Preventive Medicine* 2016, **43**(06):1124-1126, 1141. (In Chinese)

95. Yu GP: **An investigation and disposal of an outbreak of tuberculosis in Schools**. *Strait Journal of Preventive Medicine* 2009, **15**(02):40-41. (In Chinese)

96. Zhang XM, Zeng LC, Du XL, Wang XL, Han YL, Wang CJ: **An epidemiological survey of tuberculosis outbreaks in secondary schools**. *Occupation and Health* 2007, **23**(01):33-35. (In Chinese)

97. Zhang JH, Yang HT, Zhang YM, Yu AF, Chen YH, Wang F, Chen LY: **Investigation and disposal of a tuberculosis epidemic in a school**. *Zhejiang journal of preventive medicine* 2007, **19**(12):28, 31. (In Chinese)

98. Zhao DY, Wang MQ, Wang XL, Zhou LP, Luo JM, Yang CF, Wang XJ, Liu X, Pi Q: **Epidemiological investigation of a tuberculosis outbreak among senior high school students.** *Journal of Public Health and Preventive Medicine* 2013, **24**(5):90-92. (In Chinese)

99. Zhao RX: **Investigation and analysis of an outbreak of pulmonary tuberculosis in a university**. *Medical Information* 2013, **26**(10):383. (In Chinese)

100. Zhou S, Chen M: **Epidemiological survey of the breakout of pulmonary tuberculosis on a**

campus of a university in Chongqing. *Health medicine research and practice in higher institutions* 2005, **2**(02):7-10. (In Chinese)

101. Li J, Zhu XM, Shan ZL, Zhang SL, Mao LQ, Niu YY. **Investigation and analysis of a cluster of tuberculosis epidemics in a school**. *Chinese Rural Health Service Administration* 2017, **37**(8):1-1. (In Chinese)

102. Tang J. **Monitoring and management for clustering epidemics of tuberculosis colleges of Liuzhou**. *Guangxi medical university* 2017.

103. Wang ZP. **Investigation and treatment of tuberculosis epidemic in a middle school in Feng County**. *Chinese Journal of School Doctor* 2017, **31**(5): 358-359. (In Chinese)

104. Gao CM, Li HY, Ma YX, Jing ZJ, Wang LG, Qin FJ, Liu YD, Tong LB. **Epidemiological Survey of a Clustering Infection with Pulmonary Tuberculosis in a University in Jinan**. *Journal of Preventive Medicine of Chinese People's Liberation Army* 2018, **36**(10):1231-1234. (In Chinese)

105. Xu XD. **Investigation and analysis of a tuberculosis epidemic in a university in Yixing**. *Chinese Community Doctors* 2018, **34**(34):178-179,181. (In Chinese)

106. Yin CL, Zhang H, Cheng Y. **Investigation on A Cluster Outbreak of Tuberculosis Among Students in a middle school in Dazhou**. *Journal of Preventive Medicine Information* 2018, **34**(9):1177-1180. (In Chinese)

107. Ying QD. **An epidemiological survey of tuberculosis outbreaks in middle schools in Yiwu city**. *Chinese Rural Health Service Administration* 2018, **38**(7):923-924. (In Chinese)
